# ﻿*Microchiritaminor* (Gesneriaceae), a new species from north-western Vietnam

**DOI:** 10.3897/phytokeys.215.90859

**Published:** 2022-12-15

**Authors:** Zi-Bing Xin, Rui-Feng Li, Stephen Maciejewski, Long-Fei Fu, Truong Van Do, Fang Wen

**Affiliations:** 1 Guangxi Key Laboratory of Plant Conservation and Restoration Ecology in Karst Terrain, Guangxi Institute of Botany, Guangxi Zhuang Autonomous Region and Chinese Academy of Sciences, Guilin, CN-541006, China Guangxi Institute of Botany, Guangxi Zhuang Autonomous Region and Chinese Academy of Sciences Guilin China; 2 National Gesneriaceae Germplasm Resources Bank of GXIB, Gesneriad Committee of China Wild Plant Conservation Association, Gesneriad Conservation Center of China, Guilin Botanical Garden, Chinese Academy of Sciences, Guilin, CN-541006, China Guilin Botanical Garden, Chinese Academy of Sciences Guilin China; 3 College of Tourism and Landscape Architecture, Guilin University of Technology, Guilin, CN-541006, China Guilin University of Technology Guilin China; 4 The Gesneriad Society, 2030 Fitzwater Street, Philadelphia, PA. 19146-1333, USA The Gesneriad Society Philadelphia United States of America; 5 Graduate University of Science and Technology, Vietnam Academy of Science & Technology, 18th Hoang Quoc Viet Road, Cau Giay, Hanoi, Vietnam Graduate University of Science and Technology Hanoi Vietnam; 6 Vietnam National Museum of Nature, Vietnam Academy of Science & Technology, 18th Hoang Quoc Viet Road, Cau Giay, Hanoi, Vietnam Vietnam National Museum of Nature, Vietnam Academy of Science & Technology Hanoi Vietnam

**Keywords:** Didymocarpoideae, flora of Vietnam, karst limestone, *Microchirita*

## Abstract

*Microchiritaminor* (Gesneriaceae), a new species from the limestone area in Son La Province, north-western Vietnam, is described here. The new species resembles *M.hamosa*, but it differs by the combination of corolla tube shape, stamens number and the length of pistil. Detailed morphological description, together with photographic plates, information on phenology, distribution, ecology and preliminary conservation status of the new species are presented.

## ﻿Introduction

The genus *Microchirita* (C.B.Clarke) Yin Z.Wang was raised to genus from the former Chiritasect.Microchirita C.B.Clarke, according to the results of molecular phylogenetic studies (Clarke 1883; Möller et al. 2009; Wang et al. 2011; Weber et al. 2011). The genus currently comprises 39 species and six varieties (POWO 2022), including some species published in recent years (e.g. Puglisi et al. 2016; Puglisi and Middleton 2017; Rafidah 2019). The genus *Microchirita* grows exclusively in limestone karst habitats of tropical Asia, of which, Thailand is considered to be the centre of diversity of *Microchirita* with 29 species recorded (Puglisi and Middleton 2017), followed by Indonesia, Cambodia, China, India, Laos, Myanmar and Vietnam (Puglisi and Middleton 2017; Rafidah 2017; Middleton 2018; Vu 2018; Wei 2018; Fu et al. 2022; Wei et al. 2022; Wen et al. 2022).

Whilst conducting botanical explorations of limestone areas in northern Vietnam, we collected some interesting *Microchirita* specimens from one population within the Xuan Nha Nature Reserve, Son La Province, in north-western Vietnam. These unknown specimens showed similarity with *M.hamosa* (R.Br.) Yin Z.Wang (Brown 1839; Wang et al. 2011) in having unbranched stem, single basal leaf, cristate inflorescence, white corolla and hairy capsule: however, it differs from *M.hamosa* by the combination of corolla tube shape, stamens number and the length of pistil. These differences allow us to confirm that it represents a new species of *Microchirita*, which we describe here.

## ﻿Taxonomic treatment

### ﻿ Microchirita minor

Taxon classificationPlantaeLamialesGesneriaceae

Z.B.Xin, T.V.Do & F.Wen sp. nov.

C370E800-D7FA-5693-BAF1-CF5819CE44F5

urn:lsid:ipni.org:names:77309986-1

Figs 1 , 2

#### Diagnosis.

The new species is morphologically similar to *Microchiritahamosa*, but it differs from the latter in its corolla tube 5–6 mm long, with four yellow patches, each one originating at the base of each filament and reaching the throat, the throat-base diameter ratio of the corolla tube 1–1.2 (vs. corolla tube 8–15 mm long, with one yellow patch ventrally, the throat-base diameter ratio of the corolla tube 2–3); stamens 4, 2–2.5 mm long, ca. 0.3 mm in diameter (vs. stamens 2, ca. 1.5 mm long, ca. 0.1 mm in diameter); pistil 6–8 mm long (vs. ca. 16 mm long); ovary 2–3 mm long (vs. ca. 14 mm long).

**Figure 1.**
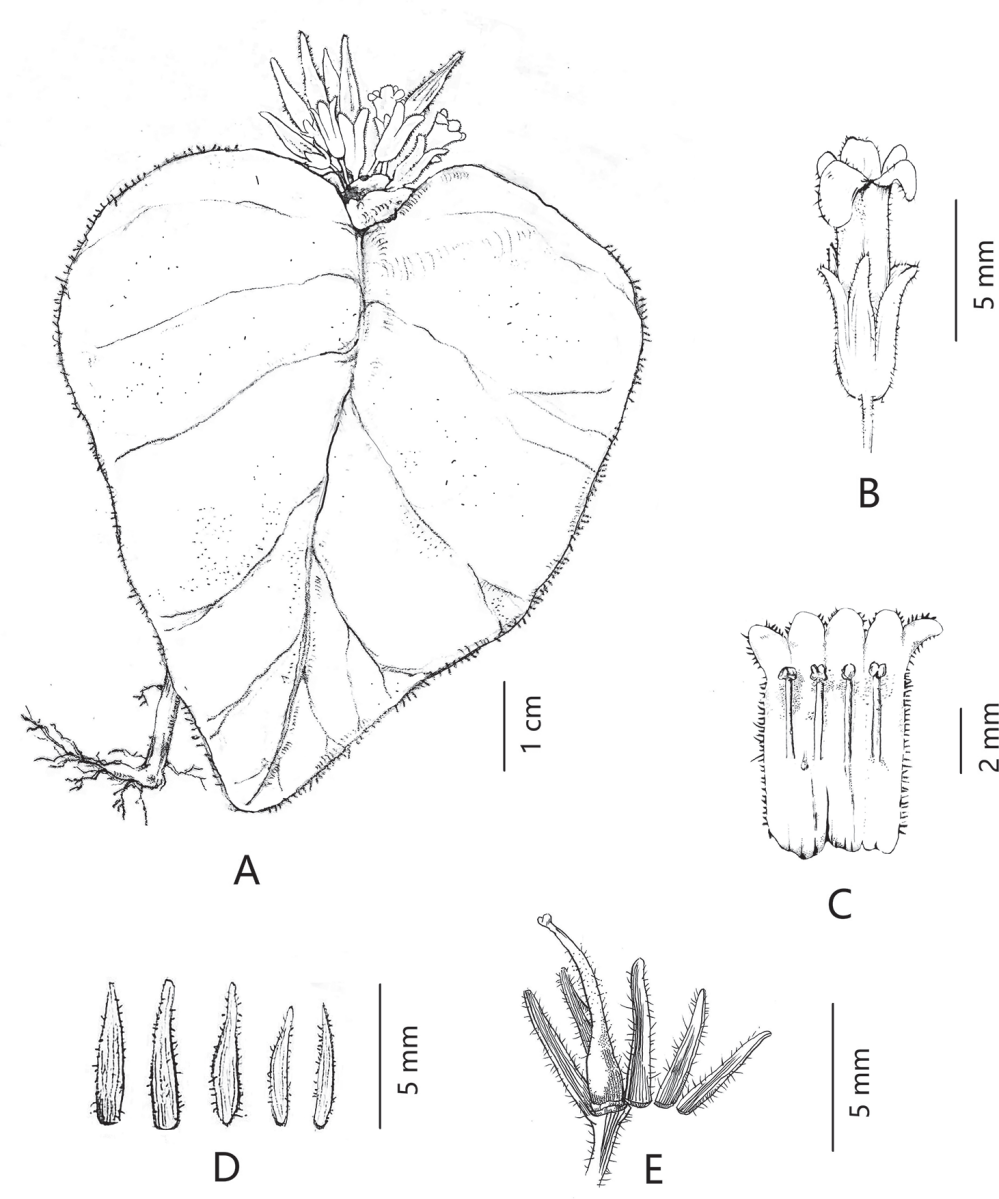
*Microchiritaminor* sp. nov. **A** habit **B** flower **C** opened corolla with stamens and staminodes; **D** abaxial surface of calyx lobes **E** pistil and calyx. Drawn by Rui-Feng Li.

#### Type.

Vietnam. Son La Province: Moc Chau District, Xuan Nha Nature Reserve, in moist crevices of the limestone cliff and mouth of limestone caves in a subtropical evergreen seasonal rainforest, 20°43'32"N, 104°40'50"E, elev. ca. 539 m, 1 November 2019, *F. Wen*, *T.V. Do*, *Z.B. Xin & S. Maciejewski VMN-CN 1231* (holotype VNMN!; isotypes IBK!, VNMN!).

#### Description.

Annual herb, up to 7 cm tall. Stems maroon green, erect or sub-erect, sparsely eglandular pubescent; unbranched. Leaves 1, rarely 3 to 5, lowermost solitary, leaves towards apex opposite; petioles 1–10 mm long, densely and shortly pubescent; blades mid-green adaxially, paler abaxially, ovate to elliptic, 1–10 cm long, 0.5–8 cm wide, base cordate broadly attenuate to obtuse, apex acute to acuminate, eglandular pubescent adaxially and abaxially, margin near entire, mid-rib impressed adaxially, prominent abaxially, lateral veins 5–7 pairs, sparsely eglandular pubescent. Inflorescences cristate, epiphyllous, 5–15-flowered; peduncles extremely short, ca. 1 mm long; bracts absent; pedicels pale green, 4–6 mm long, densely glandular and eglandular pubescent. Calyx 5, segments unequal, upper lobes 3-parted to near the base, lower lobes 2-parted to the base, the central upper lobe (alternate to the upper corolla lobes) shorter and thinner than the other lobes, pale green, lobes lanceolate, larger lobes 5–7× ca. 1 mm, smaller lobes ca. 4 × 0.6 mm, apex acuminate, margin entire, densely glandular and eglandular pubescent outside, glabrous inside. Corolla 8–10 mm long, tube white, with four yellow patches inside the tube, each one of them originating at the base of each filament and reaching the throat, corolla tube tubular, 5–6 mm long, 1.7–2.2 mm in diameter, eglandular hairy outside, glabrous inside; lobes elliptic, upper lobes 1–1.2 × 1.2–1.5 mm, lower lobes 1.2–1.5 × 1.5–2.0 mm. Stamens 4, inserted ca. 3 mm above the corolla base; filaments straight, white, glabrous, 2–2.5 mm long, ca. 0.3 mm in diameter; anthers white, papilionaceous, ca. 0.8 × 0.6 mm; staminode 1, adnate to ca. 2.5 mm above the corolla base, 0.3–0.5 mm long. Disc annular, margin entire. Pistil 6–8 mm long, densely glandular and eglandular pubescent from the base, more sparsely towards the stigma; ovary 2–3 mm long; style 3–4 mm long, eglandular pubescent, stigma elliptic, ca. 1 × 0.8 mm. Capsule green, 1.4–1.6 cm long, 1.5–2.0 mm in diameter, eglandular pubescent, straight.

**Figure 2.**
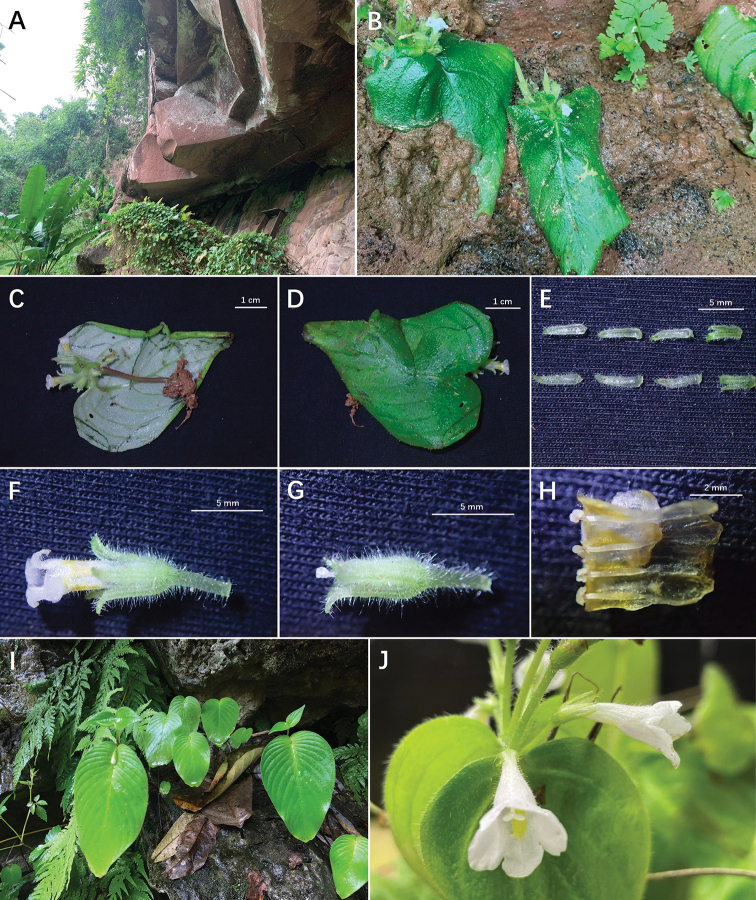
**A–H***Microchiritaminor* sp. nov. **I–J***M.hamosa***A** habitat **B** plants with flowers **C** abaxial surface of leaf blade **D** adaxial surface of leaf blades **E** calyx lobes (adaxial surface above, abaxial surface below) **F** lateral view of the flower **G** pistil and calyx **H** opened corolla with stamens and staminodes **I** habitat **J** plant with flowers. (Photos **C–H** from type material).

#### Etymology.

Latin *minor*, smaller, alluding to size of plants and flowers.

#### Phenology.

Flowering was observed from October to November. Fruiting from November to December.

#### Distribution and habitat.

The new species is currently only known from one population within Xuan Nha Nature Reserve, Moc Chau District, Son La Province, north-western Vietnam. The new species grows in moist crevices of the limestone cliff and mouth of limestone caves in a subtropical evergreen seasonal rainforest, at elevations of 530–545 m.

#### Proposed IUCN conservation status.

The new species is only known from a single population in Xuan Nha Nature Reserve, Moc Chau District, Son La Province, north-western Vietnam. This single population has no more than 1000 mature individuals, all growing on moist and shaded rocky surfaces on the cliff. They are easily disturbed by human activities as the known habitat is located in the buffer zone of the Nature Reserve and near the sugar-cane field. The species is provisionally assessed as data deficient (DD), following the IUCN Red List Categories and Criteria (IUCN 2022), because more surveys are needed.

#### Notes.

The most striking character of *Microchiritaminor* is the tiny, white corolla with four stamens. It is most easily confused with *M.hamosa*, from which it differs in the much smaller corolla, shorter corolla tube (5–6 mm long vs. 8–15 mm long), with four yellow patches inside the tube, each one of them originating at the base of each filament and reaching the throat, four larger and sturdier stamens (2–2.5 mm long, ca. 0.3 mm in diameter vs. ca. 1.5 mm long, ca. 0.1 mm in diameter) and shorter pistil (6–8 mm long vs. ca. 16 mm long). Detailed morphological comparisons of the new species with *M.hamosa* are shown in Table 1. The floral measurements of *M.hamosa* were mainly derived from Wang et al. (1990, 1998), Puglisi and Middleton (2017) and our own observations and dissection.

**Table 1.** Detailed comparison of *Microchiritaminor* and its relative *M.hamosa*.

Characters	*M.minor*	*M.hamosa*
Height of the mature plant	up to 7 cm tall	up to 25 cm tall
Peduncles	extremely short, ca. 1 mm long	up to 4 mm long
Corolla tube length	5–6 mm long	8–15 mm long
Throat-base diameter ratio of the corolla tube	1–1.2	2–3
Corolla tube colour	white with four yellow patches inside the tube, each one of them originating at the base of each filament and reaching the throat	white with one yellow patch ventrally
Stamens	four, 2–2.5 mm long, ca. 0.3 mm in diameter	two, ca. 1.5 mm long, ca. 0.1 mm in diameter
Pistil	6–8 mm long	ca. 16 mm long
Ovary	2–3 mm long	14 mm long
Stigma	ca. 1 mm long	ca. 0.2 mm long

## Supplementary Material

XML Treatment for Microchirita minor
